# Precision Chemistry for Protein Lysine Modification

**DOI:** 10.1002/chem.71193

**Published:** 2026-05-29

**Authors:** Mayu Onoda, Motomu Kanai

**Affiliations:** ^1^ Graduate School of Pharmaceutical Sciences The University of Tokyo Tokyo Japan

**Keywords:** catalysis, epigenetics, lysine, post‐translational modifications, regioselectivity

## Abstract

Lysine post‐translational modifications (PTMs) play crucial roles in regulating protein structures, interactions, and cellular signaling. Precise study of PTM functions requires homogeneous proteins with modifications at defined sites; however, selective chemical modification is challenging because multiple lysines undergo similar chemistry yet exhibit variable reactivity depending on their local environments. Early chemical strategies exploited intrinsic reactivity differences, which are effective for exposed lysines but limited for low‐reactivity or sterically hindered sites. In contrast, enzymes achieve high selectivity through substrate recognition and spatial organization. Inspired by this enzymatic principle, chemical approaches have been developed, including ligand‐directed chemistry, proximity‐enabled modification, and on‐demand generation of highly reactive electrophiles. Recent advances in ligand‐guided catalysis now enable regioselective and catalytic installation of biorelevant, authentic PTMs, providing a platform for programmable lysine modification and synthetic epigenetics.

## Graphical abstract

Selective modification of lysine residues is challenging due to their similar intrinsic reactivity. Inspired by enzymatic recognition, ligand‐guided electrophiles enable site‐selective labeling and functionalization, while ligand‐guided catalyses achieve regioselective installation of bio‐relevant post‐translational modifications. Together, these advances establish a framework for programmable protein modification and synthetic epigenetics.

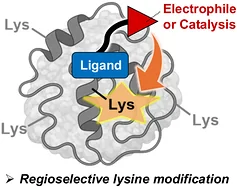

## 1 Introduction

Proteins regulate and drive diverse aspects of cellular physiology, including signal transduction, metabolism, chromatin regulation, and stress responses. While genetically encoded amino acid sequences primarily determine proteins’ higher‐order structures and functions, they are finely and dynamically modulated by chemical reactions known as post‐translational modifications (PTMs). PTMs provide a regulatory layer that extends chemical diversity beyond the amino acid sequence, enabling cells to respond rapidly and flexibly to environmental cues [1, 2, 3, 4].

Among the twenty canonical amino acids, lysine (Lys, K) occupies a uniquely privileged position as a major PTM hotspot. Lysine can undergo a broad range of covalent modifications due to the nucleophilicity of its ε‐amino group. Indeed, lysine is subject not only to classical modifications such as acetylation, methylation, and ubiquitination, but also to an expanding repertoire of acylations, including crotonylation, succinylation, and malonylation, identified by recent advances in mass spectrometry [5, 6]. These studies have established lysine PTMs as a central “chemical hub” for controlling cellular states.

Functionally, lysine modifications can modulate protein charge, disrupt or stabilize local structures, and remodel interaction networks. For example, histone lysine acetylation neutralizes positive charge, weakens DNA–histone electrostatic interactions, and is associated with chromatin relaxation and transcriptional activation [7, 8]. Lysine methylation, by contrast, does not alter the protein's charge but generates binding epitopes for reader domains such as chromodomains and Tudor domains, enabling context‐dependent transcriptional activation or repression [9]. Ubiquitination contributes to chromatin reorganization and recruitment of DNA repair machinery. Crosstalk phenomena, including ubiquitination at Lys120 in histone H2B subtype (H2BK120) that facilitates Dot1L‐mediated H3K79 methylation [10, 11], further underscores the hierarchical interplay among PTMs. Through such competing and cooperative reactions, lysine residues act as nodes that influence cellular fate.

A precise understanding of the “chemical code” of lysine PTMs requires homogeneous proteins bearing a single, site‐specific modification. Protein semi‐synthesis (PSS) [12] and genetic code expansion (GCE) [13] have emerged as strategies to meet this need. PSS enables PTM installation by chemically ligating synthetic peptides to recombinant fragments, whereas GCE allows the incorporation of modified amino acids into proteins during translation in cells. Despite their positional precision and chemical versatility, these approaches are not readily applicable to endogenous proteins in their native cellular context. Consequently, selective modification of a single lysine residue within living cells remains an unmet challenge [14].

Because lysine residues are hydrophilic and often exposed on protein surfaces, targeting a single lysine among many in a typical protein is inherently difficult. Subtle variations in local dielectric constants, electrostatic networks, hydration dynamics, and steric accessibility collectively modulate lysine p*K*_a_, deprotonation equilibria, and nucleophilicity [15]. Consequently, lysine residues do not exhibit a uniform reactivity profile, and purely chemical discrimination is fundamentally challenging. In response to these limitations, a new generation of chemical methods has begun to enable more selective modification of lysine residues in native proteins, giving rise to the conceptual framework of precision chemistry. Importantly, these concepts are being translated into practical methodologies. Site‐selective chemical modification of individual lysine residues is becoming increasingly feasible.

The purpose of this review is to organize the molecular basis underlying position‐dependent differences in the chemical reactivity of lysine residues within proteins and to provide an overview of how these insights have contributed to the development of site‐selective PTM installation technologies. First, we introduce classical studies that explained the heterogeneity of lysine reactivity through theoretical and experimental approaches, confirming the framework by which local environments influence p*K*_a_ and nucleophilicity. Next, we discuss analytical methods proposed to compare the reactivity of individual lysine residues, describing their measurement principles and scopes of application. Subsequently, we summarize strategies for site‐selective modification using chemical reagents based on the intrinsic reactivity of lysine residues and examine factors that determine selectivity in reaction design. We also explain the origins of enzyme‐mediated lysine selectivity from the perspective of substrate–enzyme spatial arrangements and structural biology findings, including cryo‐electron microscopy analyses. Based on these concepts, we present purely chemical approaches that mimic enzymatic environments by employing ligands and proximity effects. Finally, we organize currently developed PTM‐introducing catalysts according to their catalytic mechanisms, determinants of site selectivity, and intracellular applicability.

## 2 Molecular Foundations of Lysine Reactivity in Proteins

The ε‐ammonium group of lysine has an intrinsically high p*K*_a_ of approximately 10.4 in aqueous solution and is therefore predominantly protonated under physiological pH conditions [16, 17]. However, in the context of proteins, the p*K*_a_ values of lysine residues are not uniform and can vary substantially depending on their local environments. In 2019, Pahari, Sun, and Alexov released the PKAD database [18], which provides a comprehensive compilation of experimentally determined p*K*_a_ values of protein residues reported in literature and existing data sources. The database contains more than 1,500 entries derived from a wide range of wild‐type and mutant proteins. Analysis of the curated data reveals a clear environment‐dependent trend: lysine residues with large solvent‐accessible surface areas display p*K*_a_ values clustered around 9–12, close to the canonical value in water, whereas lysine residues that are partially or fully buried within the protein core, as well as lysine residues introduced by mutation into non‐native environments, display markedly depressed p*K*_a_ values. These observations underscore the heterogeneous and site‐specific dielectric properties of protein interiors.

**FIGURE 1:**
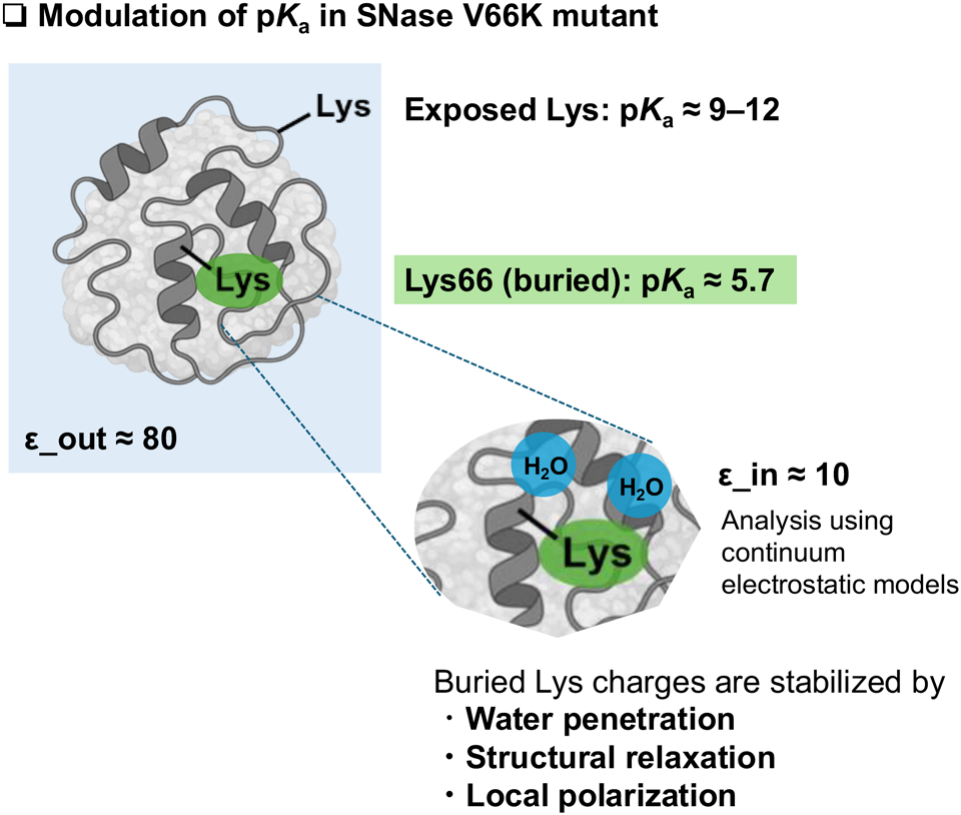
Dynamic hydration and stabilization of buried lysine residue. Lys66 in SNase is buried within the protein interior and exhibits a markedly shifted p*K*_a_ (≈ 5.7). Although the protein is embedded in bulk water, molecular dynamics simulations reveal transient water penetration and local structural relaxation near Lys‐66. These dynamic effects create locally polarized microenvironments that stabilize the buried charge without global unfolding, leading to an apparently high dielectric response.

The physical origin of p*K*_a_ depression for buried lysine residues was addressed in early mechanistic studies by Fitch, García–Moreno, and colleagues. A seminal example is their investigation of the V66K variant of staphylococcal nuclease (SNase) [19]. In this system, Val66 was selected because it is located in the hydrophobic core of the protein and is largely solvent‐inaccessible, making it an ideal site for probing the effects of burial on the p*K*_a_ of an ionizable residue. Upon substitution with lysine, the introduced Lys‐66 is deeply buried within the protein interior and exhibits an unusually low p*K*_a_ of approximately 5.7 (Figure 1). This lowered p*K*_a_ indicates that protonation of lysine is unfavorable in the protein interior, resulting in the predominance of its neutral form. The p*K*_a_ value was determined using equilibrium thermodynamic approaches, including (i) analysis of the difference in potentiometric H^+^ titration curves between the reference native protein, a hyperstable nuclease variant lacking the V66K mutation, and the corresponding V66K mutant, and (ii) linkage analysis of the pH dependence of protein stability, from which the apparent p*K*_a_ of the buried lysine residue was obtained. This substantial downward shift in p*K*_a_ is primarily attributed to the difference in electrostatic self‐energy between bulk water and the low‐dielectric environment inside the protein. This effect is commonly described as the Born energy penalty [20].

However, the magnitude of the observed p*K*_a_ shift was smaller than would be expected for transfer of a charge into a completely nonpolar environment, indicating that the charged state experiences partial stabilization within the protein matrix. Analysis using continuum electrostatic models based on the Poisson–Boltzmann equation, which partition the system into a low‐dielectric protein interior (ε_in) and a high‐dielectric aqueous environment (ε_out) [21, 22], showed that reproduction of the experimental p*K*_a_ values required assuming a relatively high effective internal dielectric constant (ε_in ≈ 10), compared with the lower values (ε_in ≈ 2–4) traditionally assigned to protein interiors surrounded by water (ε_out ≈ 80). Here, ε_in and ε_out denote the effective dielectric constants used in continuum descriptions to represent the polarizability of the protein interior and the external aqueous medium, respectively.

The authors interpreted this high apparent dielectric response as not arising from an intrinsically high static polarity of the protein interior. NMR spectroscopic and thermodynamic analyses demonstrated that ionization of Lys‐66 was not accompanied by global unfolding or major structural rearrangements of the protein. These observations suggested that large‐scale conformational changes were not the dominant source of stabilization. Rather, the data were consistent with a model in which SNase preserves its overall fold while accommodating the buried charge through localized structural relaxation and transient solvent interactions, including partial penetration of water molecules into buried regions. In this view, dynamic effects that are not explicitly treated in continuum electrostatic models, such as conformational flexibility and solvent penetration, contribute significantly to the effective stabilization of buried ionizable residues, giving rise to an apparently high dielectric response (Figure 1).

Consistent with the framework proposed by Fitch and colleagues, in which buried p*K*_a_ values reflect dynamic rather than purely static environments, Damjanović and colleagues employed extensive molecular dynamics simulations to investigate how proteins respond to ionization of sequestered residues [23]. These simulations visualized the response of internal side chains to protonation and deprotonation and showed that small positional adjustments can facilitate water ingress, leading to the formation of locally polarized microenvironments. Notably, the magnitude of the experimentally measured p*K*_a_ shift correlated with both the number of penetrating water molecules and the root‐mean‐square deviation (RMSD) of the ionized side chain, indicating that access to hydration and conformational flexibility jointly govern the free energy of the charged state. Together, these findings demonstrated that continuum descriptions relying solely on static dielectric partitioning or Born‐type energetic considerations are insufficient unless explicit solvent effects and protein dynamics are taken into account. This recognition motivated the development and application of constant‐pH molecular dynamics approaches, which treat protonation equilibria, solvent penetration, and structural relaxation in a self‐consistent manner [24, 25, 26].

A comprehensive experimental foundation for these mechanistic insights was established by Isom and colleagues, who systematically analyzed 25 SNase variants containing internally substituted lysine residues [15]. The resulting p*K*_a_ values spanned an exceptionally wide range (5.3–9.0), with positional differences alone accounting for shifts of 3–4 units. These results established that the protein interior is not a uniform low‐dielectric medium but a position‐dependent dielectric landscape in which reactivity is encoded locally by each microenvironment.

## 3 Visualizing the Chemical Reactivity Landscape of Lysine Residues

As discussed in section 2, the local p*K*_a_ of lysine residues is determined by a complex interplay of physicochemical factors, including electrostatic fields, hydration, and conformational dynamics. Yet how these molecular‐scale determinants influence the intrinsic reactivity of each lysine residue within a protein, what functional meaning such reactivity patterns carry, and how they manifest across the vast and structurally heterogeneous proteome had remained largely speculative for many years. Crucially, mass spectrometry‐based proteomics lacked any framework for measuring “reactivity”. This gap was bridged by the development of activity‐based protein profiling (ABPP) [27] and, subsequently, by isotopic tandem orthogonal proteolysis (isoTOP)‐ABPP [28, 29] developed by Cravatt and colleagues. These methods enabled direct, quantitative readout of the chemical reactivity of lysines within proteins.

**FIGURE 2:**
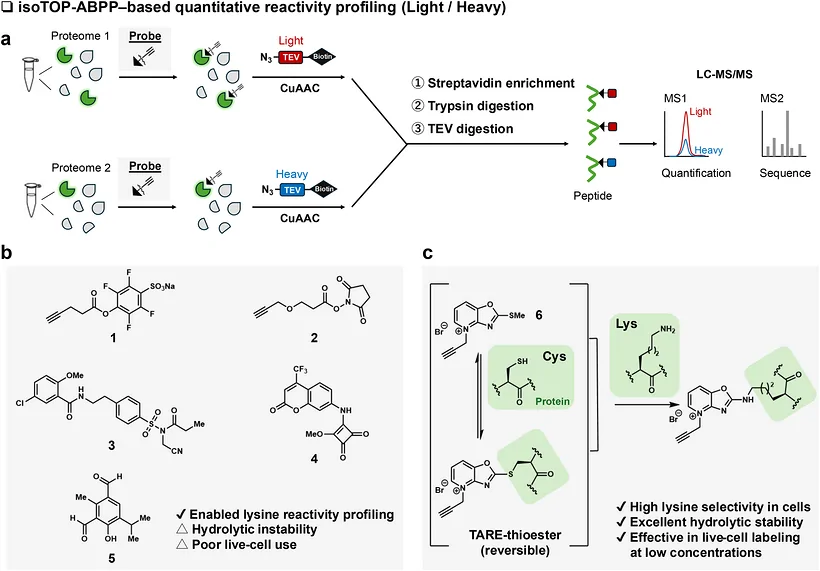
Quantitative isoTOP‐ABPP profiling of lysine reactivity. (a) Overview of quantitative isoTOP‐ABPP profiling. Proteins are treated with an alkyne‐functionalized probe and conjugated to isotopically encoded light or heavy TEV‐cleavable biotin tags via CuAAC. Probe‐modified peptides are enriched, processed, and analyzed by LC–MS/MS to determine site‐resolved lysine reactivity based on light‐to‐heavy ratios. (b) Representative lysine‐reactive probes (**1–5**): STP and NHS ester–based reagents (**1, 2**), and chemotypes from the 2021 Cravatt et al. study, including cyanomethyl acyl sulfonamides (**3**), squarates (**4**), and dicarboxaldehydes (**5**), which exhibit chemotype‐dependent lysine engagement across the proteome. (c) Structure and reaction mechanism of a cell‐compatible tunable amine‐reactive electrophile (TARE) probe (**6**). TAREs engage cysteine residues reversibly to form a transient thioester intermediate, which subsequently undergoes irreversible reaction with lysine ε‐amino groups, conferring high lysine selectivity. This two‐step mechanism provides excellent hydrolytic stability and enables efficient lysine labeling in living cells at low probe concentrations.

IsoTOP‐ABPP integrates residue‐selective chemical labeling, click chemistry, biotin enrichment, sequential protease digestion, and isotope‐encoded mass spectrometry into a unified platform (Figure 2a). Upon treatment of cell lysates with an alkyne‐functionalized electrophilic probe, labeling proceeds preferentially at nucleophilic amino acid residues reflecting their local microenvironment. Subsequent installation of an azide‐modified TEV‐biotin tag via copper‐catalyzed azide alkyne cycloaddition (CuAAC), streptavidin enrichment, tryptic digestion, and TEV cleavage yields peptide fragments carrying probe‐modified residues. LC–MS/MS analysis then enables quantitative, site‐resolved measurement of probe reactivity across the proteome.

Although originally developed for cysteine profiling [28], this approach was extended to lysine residues with the introduction of the sulfotetrafluorophenyl (STP) ester probe in 2017 (**1**), enabling mapping of more than 9,000 lysine sites in human cells [30]. This dataset revealed several hundred highly reactive lysine residues, which were not randomly distributed but were instead enriched in functionally annotated regions, including enzyme active sites, allosteric sites, protein–protein interaction interfaces, ligand‐binding pockets, and transcriptional regulatory complexes. Around the same period, *N*‐hydroxysuccinimide (NHS) ester–based probes were also reported (**2**). These probes react with lysine residues as well as other nucleophilic amino acids, including serine, threonine, and tyrosine, and have been applied to map reactive hotspots and local protein microenvironments [31].

In 2021, Cravatt and coworkers reported a structurally diverse library of approximately 180 amine‐reactive compounds and applied isoTOP‐ABPP to systematically assess lysine ligandability [32]. This study evaluated multiple chemotypes, including cyanomethyl acyl sulfonamides (e.g., **3**) and squarates (e.g., **4**), and demonstrated that lysine engagement is dependent on the chemical structure of the compounds, revealing distinct structure–activity relationships (SARs). Among these, dicarboxaldehyde compounds (e.g., **5**) exhibited broad reactivity, engaging over 200 lysine residues across the proteome and functioning as “scout fragments” for proteome‐wide assessment of lysine reactivity. These aldehydes form reversible imine adducts with lysine ε‐amino groups, enabling broad mapping of interaction profiles. In contrast, more selective chemotypes such as **3** and **4** were found to react with different lysine residues across the proteome. In particular, **3** was found to engage IFIT5 at K150, whereas **4** engaged XRCC6 at K351.

However, STP‐ and NHS ester–based probes are prone to hydrolysis and exhibit limited stability and selectivity in cellular environments, and thus have primarily been applied under cell lysate conditions. In addition, although several compounds from the 2021 amine‐reactive library, including cyanomethyl acyl sulfonamides **3**, were shown to engage target proteins in cellular systems, these compounds were not specifically optimized for intracellular stability or selectivity and were primarily developed for systematic exploration of lysine ligandability. As a result, their application in live‐cell contexts remained limited.

These limitations were addressed by the introduction of Tunable Amine‐Reactive Electrophiles (TAREs) [33], which enabled practical lysine‐selective covalent labeling in living cells. The defining feature of TARE chemistry is a deliberately engineered two‐step mechanism that decouples the initial point of attack from the final site of modification. In the first step, TAREs react reversibly with cysteine to form a labile S‐adduct. In the second step, a new electrophilic center is generated that reacts irreversibly with the ε‐amino group of lysine to give a stable covalent adduct. Thus, cysteine functions only as a transient entry point, whereas the final and persistent modification occurs exclusively on lysine (Figure 2c). This reversible cysteine engagement followed by irreversible lysine functionalization underpins the unique lysine selectivity of TAREs.

Chemoproteomic analyses support this design: probe **6** (Figure 2c) modified thousands of lysine residues across the proteome, whereas cysteine modifications were observed only at trace levels. This behavior was consistent with the intrinsic instability of the S‐adducts and with DFT calculations indicating that reactions with thiolates are reversible, whereas reactions with amines are irreversible and thermodynamically favored. Importantly, TAREs enabled efficient lysine‐selective labeling directly in living cells. Although conventional STP‐based probes can label proteins in live cells, they require relatively high concentrations (5–100 µM), owing to rapid hydrolysis and substantial off‐target reactivity. In contrast, probe **6** exhibits excellent hydrolytic stability, very low cytotoxicity, and efficient cellular uptake, enabling robust intracellular labeling within minutes at concentrations as low as 100 nM. These two‐to‐three orders of magnitude reductions in the effective labeling concentration highlight fundamental differences between STP‐ and TARE‐based probes in chemical stability, target selectivity, and compatibility with the living cellular environment.

## 4 Reaction‐Encoded Strategies for Achieving Regioselectivity

### 4.1 Kinetic Regioselectivity: Exploiting Intrinsic Reaction‐Rate Hierarchies

As it has become increasingly clear that lysines display fundamentally heterogeneous intrinsic reactivity, a central question emerges: how can a single lysine be selectively modified within such a chemically diverse ensemble? The principle of kinetic lysine selectivity provides a solution to this question, in which regioselectivity arises solely from the hierarchy of intrinsic reaction rates encoded on the protein surface.

Chen and colleagues demonstrated this concept using active esters, particularly NHS esters [34]. In their approach, proteins were briefly exposed to an NHS ester under mild conditions, after which the reaction was rapidly quenched. Within this narrow temporal window, only the fastest‐reacting lysine forms a covalent adduct, whereas slower‐reacting residues fail to reach the transition state before the reaction is terminated. Consequently, even proteins containing numerous lysines undergo regioselective modification at the single residue that is most kinetically privileged according to their innate reactivity landscape.

**FIGURE 3:**
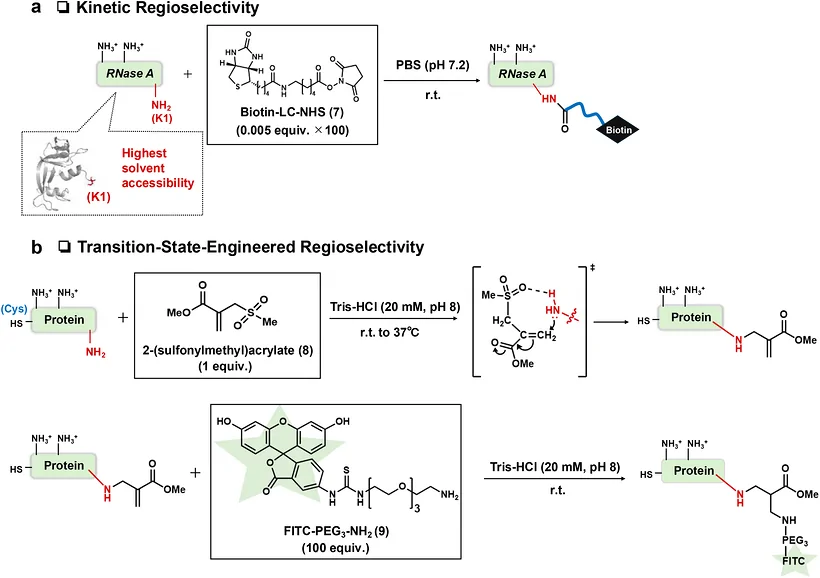
Reaction‐encoded strategies for lysine regioselectivity. (a) Kinetic control using an activated ester **7** enables selective modification of the most rapidly reacting lysine residue under brief, substoichiometric labeling conditions, exemplified by Lys1 of RNase A. (b) Sulfonyl acrylates **8** achieve lysine regioselectivity by decoding local p*K*_a_ differences, preferentially modifying low‐p*K*_a_ lysines in hydrophobic microenvironments through transition‐state stabilization, while excluding high‐p*K*_a_ lysines and cysteine residues. The resulting acrylate adduct serves as a versatile handle for subsequent installation of functional tags, including fluorescent probes such as FITC.

This principle is exemplified by detailed studies on RNase A, which contains ten surface lysines (Figure 3a). Under kinetic labeling conditions, the authors employed biotin‐LC‐NHS (a long‐chain biotinylated NHS ester) (**7**) at only 0.5 equiv., divided into 100 portions and added stepwise over 100 min. This protocol ensured that RNase A remained in excess at all times, such that the most rapidly reacting lysine residue immediately consumed each increment of reagent. Under these conditions, >90% single‐site modification was achieved, confirming that the fastest‐reacting lysine was consistently captured throughout the reaction. Notably, the modified lysine residue (K1) in RNase A corresponds to the site exhibiting the highest solvent accessibility, as determined by crystallographic analysis. The generality of this kinetic strategy is evident. Optimized activated‐ester reagents enabled >90% single‐site labeling across diverse folded proteins, including RNase A, lysozyme C, and the peptide hormone somatostatin, without perturbing native tertiary structure, as the reaction is terminated before substantial conformational change occurs.

### 4.2 Transition‐State–Engineered Regioselectivity: Sulfonyl Acrylates as p*K*_a_‐Decoding Electrophiles

NHS‐based kinetic selection inherently requires substoichiometric electrophile conditions, precluding full conversion by design. Although this strategy is effective for identifying the most reactive lysine residue within a protein, it limits broader synthetic applications. To overcome this limitation, Matos and co‐workers developed sulfonyl acrylates as electrophiles that achieve selectivity through transition‐state stabilization rather than reagent stoichiometry [35]. In this approach, selective modification under equimolar conditions arises from preferential stabilization of the transition state associated with nucleophilic addition by low‐p*K*_a_ lysine residues.

This strategy exploits the intrinsic p*K*_a_ heterogeneity of lysine side chains. Lysines located in hydrophobic microenvironments tend to exhibit lower p*K*_a_ values and therefore exist more frequently in the neutral NH_2_ form, thereby enhancing nucleophilicity. Quantum chemical analysis of Michael acceptors identified 2‐(sulfonylmethyl)acrylate (**8**) as possessing a particularly low activation barrier (Figure 3b). This enhanced reactivity was attributed to a transition‐state hydrogen bond between the sulfone moiety and the neutral NH_2_ group of low‐p*K*_a_ lysines, which stabilizes charge development during nucleophilic addition. In contrast, high‐p*K*_a_ lysines remain predominantly protonated (NH_3_^+^), requiring prior deprotonation and lacking an available lone pair, resulting in substantially reduced reactivity. The reaction is performed at pH 8.0, a condition that is critical for chemoselectivity. Under these conditions, cysteine residues exist predominantly in the neutral thiol (S─H) form, while formation of the more reactive thiolate species (S^−^) is minimized. In addition, the weak polarity of the S─H bond limits its ability to participate in hydrogen‐bond donation, thereby preventing effective transition‐state stabilization. Consequently, the cysteine‐mediated reaction pathway is energetically disfavored, consistent with quantum chemical calculations and potential energy surface analyses. Sulfonyl acrylates, therefore, achieve selectivity by preferentially reacting with lysine residues capable of forming stabilizing transition‐state hydrogen‐bond interactions while retaining the reactive nitrogen atom's lone pair during nucleophilic addition. Furthermore, the resulting acrylate adduct serves as a versatile platform for the installation of functional moieties, such as FITC–PEG_3_–NH_2_ (**9**), thereby expanding the utility of this chemistry for bioconjugation applications (Figure 3b) [35].

The robustness and generality of this strategy were further demonstrated across a representative panel of structurally and functionally diverse proteins, including human serum albumin, lysozyme, the C2A domain of synaptotagmin‐I, annexin V, and the therapeutic IgG antibody trastuzumab. In every case, sulfonyl acrylates achieved residue‐level selectivity, consistently modifying the lysine predicted to have the lowest p*K*_a_, while maintaining high chemoselectivity in the presence of free cysteine residues and full compatibility with folded protein architectures ranging from small enzymes to full‐length antibodies.

Nonetheless, kinetic regioselectivity and transition‐state–engineered regioselectivity approaches share a common limitation: both inherently access only the most reactive lysines within a protein. Sterically shielded lysines, high‐p*K*_a_ lysines, and residues located in chemically unprivileged environments remain largely inaccessible. Achieving truly general and site‐diverse lysine modification will therefore require strategies that transcend intrinsic nucleophilicity. The most instructive blueprint comes from enzymes, which override substrate reactivity hierarchies through integrated sequence recognition, binding geometry, and microenvironmental engineering to direct reactivity toward a specific lysine. The next section examines this enzymatic logic and discusses how these principles may guide the design of next‐generation chemical strategies capable of targeting any lysine within the proteome.

## 5 Regioselective Lysine Modifications in Nature: Precision Substrate Recognition by Enzymes

Protein‐modifying enzymes do not simply read out the intrinsic reactivities of lysines in a passive manner. Instead, they embed themselves within the substrates, precisely controlling binding position, substrate presentation, and spatial orientation, thereby achieving extraordinary levels of regioselectivity. Histone‐modifying enzymes RNF168, BRCA1/BARD1, and Dot1L represent three paradigmatic examples. Despite acting on the same nucleosomal histones, each enzyme targets a different lysine residue through distinct structural logic.

Genetic information is stored in DNA. In cells, DNA exists as complexes with polycationic proteins, called histones, forming chromatin. Mononucleosome is the minimum unit of chromatin, which contains >50 lysine residues. These lysine residues undergo various chemical modifications, including acetylation, malonylation, methylation, and ubiquitination. Histone modification patterns are derived from combinations of various reaction types and positions, together constituting the epigenome [5, 36]. The epigenome controls diverse chromatin functions, including gene regulation, transcription, and DNA damage repair [36, 37]. Therefore, chromatin histones are a particularly intriguing substrate for examining the reactivity of each lysine residue within a protein.

RNF168 and BRCA1/BARD1 are E3 ubiquitin ligases that utilize the same E2 enzyme, UbcH5c, yet regioselectively ubiquitinate distinct lysine residues on the conformationally flexible tail region of histone H2A [38, 39, 40, 41]. RNF168 primarily targets Lys13 and Lys15 located on the *N*‐terminal tail, whereas BRCA1/BARD1 selectively modifies Lys125, Lys127, and Lys129 within the C‐terminal tail. Although both E3 ligases bind to the same region of a nucleosome, acidic patch, via their RING domains using a conserved arginine anchor motif, recent cryo‐EM studies have revealed that their divergent lysine selectivities arise from fundamentally different pre‐reaction geometries formed on the nucleosome [40, 41].

**FIGURE 4:**
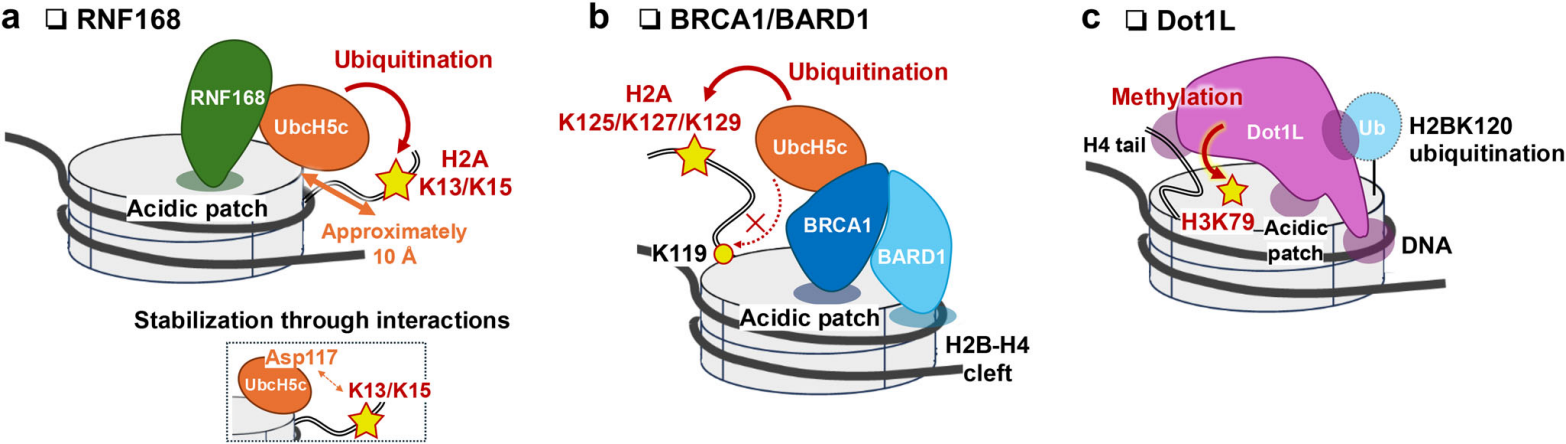
Enzyme‐driven lysine regioselectivity on nucleosomes. (a) RNF168 binds the nucleosome acidic patch and positions UbcH5c close to the H2A *N*‐terminal tail, enabling selective ubiquitination of K13 and K15 through spatial proximity and E2‐mediated gating. (b) BRCA1/BARD1 engages the acidic patch and the H2B–H4 cleft, lifting UbcH5c away from the nucleosome surface and allowing selective ubiquitination of the flexible H2A *C*‐terminal lysines (K125, K127, K129) while excluding K119. (c) Dot1L binds the nucleosome via multivalent interactions, and H2BK120 ubiquitination stabilizes a catalytically competent geometry that enables highly selective methylation of the globular lysine H3K79.

In the RNF168 complex with a mononucleosome, UbcH5c was positioned in close proximity to the nucleosome surface, such that its active site was oriented toward the *N*‐terminal tail of H2A. Cryo‐EM structures showed that the Cα atoms of H2AK13 and K15 were approximately 10 Å from the catalytic cysteine (Cys85) of UbcH5c, a separation compatible with efficient ubiquitin transfer given side‐chain flexibility [40]. In addition, Asp117 of UbcH5c functioned as a gating residue that regulates substrate lysine entry into the active site, thereby stabilizing this pre‐attack conformation. Consequently, selective ubiquitination of H2AK13/K15 by RNF168 was achieved through a combination of spatial proximity and E2‐mediated gating (Figure 4a).

By contrast, BRCA1/BARD1 adopts a markedly different geometric arrangement. BRCA1/BARD1 functions as a heterodimeric E3 complex in which the BRCA1 RING binds the nucleosome acidic patch, while its partner BARD1 forms a novel and specific interface with the H2B/H4 cleft (Figure 4b). This interaction constrains BARD1 close to the histone surface and induces an upward tilt of the E2‐binding interface on BRCA1. As a result, UbcH5c is lifted away from the nucleosome surface, positioning its active site at a greater distance from the histone core [41]. In this configuration, the intrinsically disordered C‐terminal tail of H2A retains high conformational freedom, allowing Lys125, Lys127, and Lys129 to reach the elevated UbcH5c active site and undergo efficient ubiquitin transfer. In contrast, Lys119—although proximal in sequence—is located within the histone fold domain and was structurally constrained on the nucleosome surface. This residue is therefore unable to access the repositioned UbcH5c active site, rendering ubiquitination at Lys119 unfavorable. Thus, lysine selectivity in the BRCA1/BARD1 system emerges from a flexible geometry that still favored the substrate's lysine with sufficient reach to the catalytic site of the enzyme.

Dot1L is the sole methyltransferase responsible for methylation of histone H3 at Lys79 and exhibits exceptionally strict regioselectivity [11, 42, 43]. Methylation at Lys79 in H3, particularly trimethylation (H3K79Me_3_), promotes gene activation and is frequently associated with leukemogenesis [44]. Ubiquitination at Lys120 in H2B (H2BK120Ub) enhances Dot1L enzymatic activity through crosstalk of epigenetic marks [43]. The origin of the regioselectivity of Dot1L is fundamentally distinct from that of many other histone‐modifying enzymes that target lysines at flexible histone tails, including RNF168 and BRCA1/BARD1. H3K79 is a rigid, so‐called “globular lysine” embedded within the histone fold domain, occupying a position that is physically inaccessible unless the enzyme is oriented with high precision. Consequently, residue selectivity by Dot1L is not dictated by dynamic discrimination of the lysine side chain, but instead by the formation of a highly defined geometric arrangement on the nucleosome.

Cryo‐EM structural analyses revealed that Dot1L binds to the nucleosome surface through multivalent interactions (Figure 4c). The primary contact sites included the histone H4 tail and the nucleosome acidic patch, with Arg282 of Dot1L functioning as a key anchoring residue at the acidic patch. Importantly, these interactions are observed irrespective of the ubiquitination status of H2BK120, indicating that Dot1L fundamentally recognizes the same nucleosomal surface in both modified and unmodified contexts [42]. H2BK120Ub instead suppresses conformational fluctuations of Dot1L in the bound state. Structural and mutational analyses suggest that ubiquitin reduces the conformational sampling space of Dot1L, thereby stabilizing a catalytically competent geometry in which the active site is precisely aligned with H3K79. This geometric constraint enables Dot1L to catalyze methyl transfer exclusively to the globular lysine H3K79 [42].

Considered together with RNF168 and BRCA1/BARD1, Dot1L highlights a unifying principle of lysine regioselectivity. Selectivity is not governed by the intrinsic chemical reactivity of the lysine ε‐amine, but rather emerges from the coordinated integration of structural determinants, including protein‐binding topology, anchor‐point interactions, catalytic orientation and distance, encounter‐complex dynamics, and auxiliary post‐translational modifications such as H2BK120Ub. Through distinct three‐dimensional binding logics, nature funnels catalytic activity toward a single lysine residue while effectively excluding all others.

If one seeks to recapitulate such exquisite positional control in a purely chemical, non‐enzymatic context, the implication is clear: selectivity must be constructed, not discovered. Chemical strategies cannot rely solely on inherent lysine reactivity hierarchies, but must instead impose spatial constraints analogous to those encoded by enzyme architectures. The most general and programmable means of achieving this is proximity: positioning a reactive moiety in the vicinity of a designated lysine by tethering it to a ligand that recognizes a specific epitope of a target protein. Acting as the chemical analog of an enzymatic recognition domain, the ligand binds to the protein and, together with the linker, controls the orientation, distance, and local concentration of the appended reactive unit. Ligand‐guided proximity provides a foundation for achieving regioselective lysine modification beyond intrinsically most reactive sites.

## 6 Evolution of Ligand‐Directed Chemistry for Selective Modification of Native Proteins

The intracellular environment is highly crowded and heterogeneous, making the selective chemical modification of endogenous proteins a longstanding challenge. A conceptual breakthrough was introduced by Hamachi, who proposed a clear principle: a reactive functionality does not require high reactivity; rather, it can simply be delivered to its target through a high‐affinity, high‐specificity ligand. This insight established ligand‐directed chemistry (LD chemistry) as a new framework for introducing covalent conjugation to endogenous proteins without relying on genetic engineering, enzyme fusion, or overexpression [45, 46, 47, 48, 49, 50, 51].

The central idea of LD chemistry is to engineer the reactive moiety to be deliberately weak, ensuring that it remains unreactive toward myriad off‐target nucleophiles in cells, without binding. Its reactivity becomes meaningful only when ligand binding forces the electrophilic warhead into nanometer‐scale proximity to a specific amino acid residue. This “proximity‐enabled reactivity” enables covalent capture exclusively near the ligand‐bound target, effectively overriding the intrinsic reactivity hierarchy of cellular proteins. LD chemistry has progressively evolved through innovations such as affinity‐assisted acyl transfer [48, 49, 50, 51] and ultrafast ligand‐directed *N*‐acylation [52, 53, 54, 55] yielding increasingly precise and rapidly reacting lysine‐targeting strategies. These methods were originally developed for applications such as live‐cell imaging or covalent ligand discovery. They offer crucial insights into the design principles required for chemically directing reactivity toward a specific lysine residue.

### 6.1 Affinity‐Assisted Acylation: The LDAI Strategy

**FIGURE 5:**
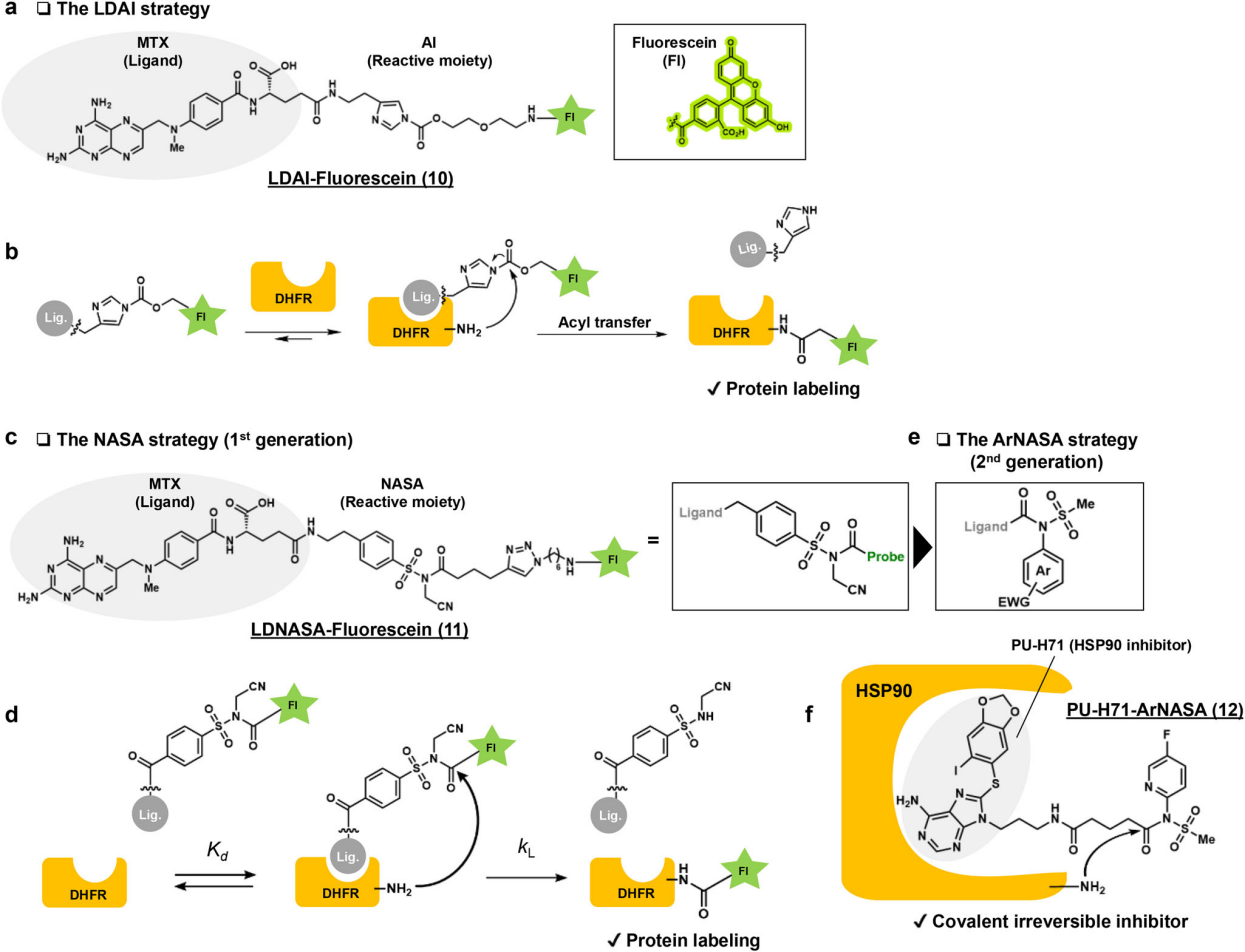
Ligand‐directed chemistry for selective lysine modification of native proteins. (a, b) Concept and mechanism of ligand‐directed acyl imidazole (LDAI) chemistry. A high‐affinity ligand (e.g., MTX) delivers an acyl imidazole warhead to a target protein, enabling proximity‐driven and site‐selective lysine modification. Ligand binding positions the reactive warhead near a specific lysine residue at the entrance of the DHFR binding pocket, resulting in single‐site labeling. (c, d) Concept and mechanism of ligand‐directed *N*‐acyl‐*N*‐alkyl sulfonamide (NASA) chemistry. A ligand‐directed NASA reagent, in which the weakly reactive NASA warhead is tethered to MTX, enables proximity‐induced lysine acylation under biological conditions. Ligand binding promotes favorable local concentration and orientation of the reactive partners, markedly accelerating acyl transfer and achieving enzyme‐like effective second‐order rate constants (*k*_L_/*K*_d_). (e) Second‐generation ArNASA electrophiles. Electronic tuning of the sulfonamide scaffold improves aqueous stability and chemoselectivity while preserving ultrafast proximity‐enabled reactivity. (f) Design of covalent inhibitors based on ArNASA chemistry. An ArNASA warhead tethered to an HSP90‐directed ligand enables irreversible and site‐selective lysine modification, resulting in efficient covalent inhibition of HSP90.

Ojida, Hamachi, and colleagues advanced the concept of LD chemistry by developing a ligand‐directed acyl imidazole (LDAI) platform, in which a moderately reactive alkyloxyacyl imidazole is tethered to a high‐affinity ligand [51]. Dihydrofolate reductase (DHFR), a soluble endogenous protein, was employed as a model system. Using LDAI‐Fluorescein (**10**, Figure 5a), which consists of methotrexate (MTX) as the anchoring ligand, an alkoxyacyl imidazole (AI) as the reactive moiety, and a fluorescent tag as the detection handle, selective labeling of a single lysine residue (K32) located at the entrance of the ligand‐binding pocket of DHFR was achieved under in vitro conditions (Figure 5b). The molecule is designed to dissociate the ligand moiety after covalent modification. Because the ligand is a substrate analog, the protein function would otherwise be abolished. This LDAI strategy was further extended to an endogenous membrane protein, the folate receptor (FR). Application of the LDAI reagent enabled selective labeling of endogenous FR under live‐cell conditions, allowing direct visualization of FR on the live cell membrane. Importantly, FR retained its native ligand‐binding capability and endocytic function following covalent labeling, which can be attributed to the fact that the ligand is released after modification and therefore does not remain bound to the active site. Thus, LDAI represents more than a simple covalent labeling method; it functions as a chemical biosensor that enables visualization and tracking of endogenous membrane proteins in live cells while preserving their biological function.

### 6.2 Ultrafast Ligand‐Directed Acylation: The NASA Strategy

LDAI chemistry represented a major advance in site‐selective labeling of endogenous proteins; however, reaction rates in living cells remained insufficient, often requiring several hours to a full day to achieve appreciable labeling [48, 49, 50, 51]. This limitation highlighted the need for a new class of proximity‐gated electrophiles that react exclusively when positioned near a target residue while operating at substantially higher speed. In response to this demand, *N*‐acyl‐*N*‐alkyl sulfonamides (NASAs) were developed (Figure 5c) [52, 53, 54, 55, 56, 57].

The defining feature of NASA electrophiles is their pronounced biphasic reactivity: they are essentially inert in bulk aqueous solution, yet become highly reactive upon proximity to a target lysine ε‐amine. The *N*‐acyl carbonyl of NASA is intrinsically resistant to nucleophilic attack because of amide‐like resonance delocalization of the nitrogen lone pair into the carbonyl group, which decreases the electrophilicity of the carbonyl carbon and suppresses background reactivity. However, ligand binding positions the warhead within a few angstroms of the lysine side chain. Under these conditions, the effective local concentration of the electrophile around the target lysine increases dramatically, while ligand‐induced conformational restriction promotes a favorable reaction geometry between the reactive partners. Together, these local concentration and orientational effects stabilize the transition state and markedly accelerate nucleophilic acyl substitution with sulfonamide departure [56, 57].

Kinetic analyses further support this proximity‐driven reaction design [53]. In ligand‐directed NASA (LDNASA) chemistry, the effective second‐order rate constant for protein labeling (*k*_L_/*K*_d,_ shown in Figure 5d) reaches approximately 10^4^ M^−^^1^ s^−^^1^, placing it on par with many enzymatic reactions and among the fastest bioorthogonal transformations, such as inverse electron‐demand Diels–Alder (IEDDA) [58] cycloadditions. Thus, NASA chemistry achieves enzyme‐like or click‐like reaction rates under biological conditions.

First‐generation NASA [53] clearly demonstrated the principle of proximity‐driven reactivity. Replacement of the acyl imidazole (AI) warhead in LDAI with NASA led to a marked increase in reaction rate, enabling rapid protein labeling. Indeed, intracellular labeling of the folate receptor (FR) in KB cells was achieved within 30 min using LDNASA‐Fluorescein (**11**, Figure 5c). In contrast, conventional LDAI reagents produced little to no detectable signal over the same time frame [53, 54]. However, the high electrophilicity imparted by the cyanomethyl substituent compromised aqueous stability and resulted in increased background reactivity.

To address these issues, second‐generation ArNASAs were developed based on a design strategy that emphasizes precise tuning of electronic structure rather than indiscriminately increasing intrinsic reactivity (Figure 5e) [54, 55]. Introduction of an aromatic substituent onto the sulfonamide nitrogen electronically stabilizes the N─S bond, resulting in markedly improved aqueous stability and selectivity, while preserving the rapid reactivity enabled by ligand‐induced proximity effects. The effectiveness of ArNASAs was clearly demonstrated in studies targeting the molecular chaperone heat shock protein 90 (HSP90). ArNASA reagents tethered to the HSP90‐selective ligand PU‐H71 (**12**, Figure 5f) functioned as irreversible covalent inhibitors, achieving site‐selective acylation of a single lysine residue (K58) on HSP90 with excellent kinetics and high efficiency. Furthermore, docking simulations of the covalent modification process suggested that interactions between the pyridine‐containing ArNASA warhead and the reactive lysine residue promote favorable orientational preorganization of the reactive partners, thereby enhancing the local proximity effect and facilitating the acyl transfer reaction [54, 55].

Collectively, ArNASA electrophiles satisfy all essential requirements for LD chemistry in complex biological environments, including aqueous stability, minimal nonspecific reactivity, ultrafast proximity‐induced acyl transfer, and precise single‐lysine selectivity. As such, they now represent a highly powerful electrophile platform not only for intracellular protein labeling but also for the design of covalent inhibitors.

### 6.3 Bioorthogonal Click‐and‐Release Generation of Isocyanates via SPICC Chemistry

A key concept in LD chemistry is that molecular recognition can be used to spatially control chemical reactivity. In earlier strategies, electrophilic warheads with intrinsic reactivity were incorporated into molecular scaffolds, and their reactivity was selectively unmasked through ligand‐induced proximity effects. More recently, “on‐demand” design strategies have been developed in which reactivity is suppressed under standard conditions and selectively activated in response to specific environments or triggers. In these approaches, highly reactive intermediates are generated in the vicinity of the target, thereby reducing non‐specific background reactions and unintended hydrolytic decomposition, while enabling the use of otherwise highly reactive chemical species.

**FIGURE 6:**
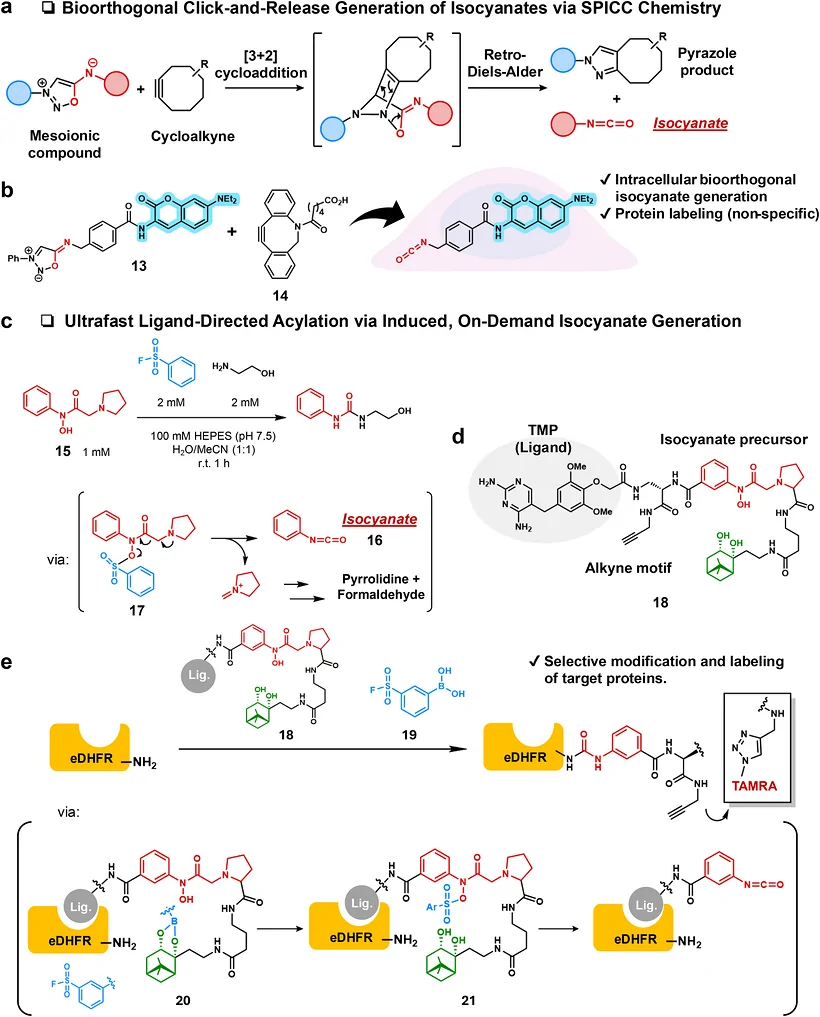
Isocyanate‐based strategies for bioorthogonal labeling and protein modification. (a) Schematic representation of the SPICC (strain‐promoted iminosydnone–cyclooctyne cycloaddition) reaction. Iminosydnones undergo a copper‐free [3+2] cycloaddition with strained cyclooctynes, followed by fragmentation to yield a pyrazole product with concomitant generation of a reactive isocyanate species. (b) Cellular application of SPICC‐based isocyanate release. A fluorescent iminosydnone probe **13** reacts with a strained cyclooctyne **14** in living cells, leading to intracellular isocyanate generation and subsequent protein labeling. (c) Mechanism of on‐demand isocyanate generation. A hydroxamic acid–pyrrolidine conjugate **15** reacts with a sulfonyl fluoride to form an *O*‐sulfonylated hydroxamate intermediate, which subsequently undergoes elimination to generate a highly reactive isocyanate **16**. (d, e) Ligand‐directed protein modification using a trimethoprim (TMP)‐tethered isocyanate precursor **18**, enabling selective targeting of *Escherichia coli* dihydrofolate reductase (eDHFR). Proximity effects mediated by boronate ester formation promote localized isocyanate generation and lysine acylation under physiological conditions, followed by alkyne‐based click functionalization (e.g., TAMRA labeling).

In 2017, Taran and coworkers reported a bioorthogonal click‐and‐release reaction termed SPICC (strain‐promoted iminosydnone–cyclooctyne cycloaddition) [59]. In this reaction, iminosydnones react with cyclooctynes via a [3+2] cycloaddition, followed by a fragmentation process that yields a pyrazole product accompanied by the release of an isocyanate (Figure 6a). Isocyanates are highly electrophilic species that react rapidly with nucleophiles such as amines and thiols under physiological conditions, making them useful intermediates for chemical modification in biological environments.

Subsequently, iminosydnone derivatives bearing alkyl or aryl substituents at the 6‐*N* position were developed [60]. These compounds were shown to exhibit lower resonance stabilization energies compared to previously reported analogs. This suggests a reduced contribution of delocalized mesoionic resonance structures, which may account for their increased reactivity. These mesoionic compounds react with strained cycloalkynes under physiological, copper‐free conditions, and function as caged precursors for isocyanate release. Reported rate constants depend on structure and reaction conditions, reaching values of up to approximately 1000 M^−^^1^ s^−^^1^. In cellular experiments, a fluorescently labeled mesoionic compound (**13**, Figure 6b) was applied to living cells, and intracellular protein labeling was observed in the presence of strained cyclooctyne reagents (**14**) in systems designed to release aryl isocyanates. This labeling was shown to depend on intracellular isocyanate formation, as direct addition of isocyanates did not result in comparable labeling efficiency. These results indicate that the SPICC reaction enables the generation of isocyanate electrophiles in living cells under bioorthogonal conditions.

### 6.4 Ultrafast Ligand‐Directed Acylation via Induced, On‐Demand Isocyanate Generation

Building on Taran's concept of the in situ generation of reactive intermediates under bioorthogonal conditions, Yamanashi, Kanai, and coworkers reported in 2025 a ligand‐directed strategy that enables on‐demand isocyanate generation from stable precursor molecules through activation by an external reagent [61]. This study extends the conceptual framework of SPICC chemistry to ligand‐directed systems, while employing a distinct activation mechanism.

A key feature of this strategy is the use of chemically stable precursor molecules. The authors discovered that a stable hydroxamic acid–pyrrolidine conjugate **15** is rapidly converted into isocyanate **16** upon treatment with a sulfonyl fluoride, and mechanistic analyses revealed that the reaction proceeds via an *O*‐sulfonylated hydroxamate intermediate **17**, followed by elimination to generate the isocyanate (Figure 6c). This insight led to a molecular design in which a highly reactive electrophile is generated on demand from a stable precursor.

Reaction control was further refined by introducing reversible boronate ester formation between a nopol diol moiety and boronic acid‐bearing sulfonyl fluoride **19**. In this design, the nopol diol unit transiently captures the sulfonyl fluoride via boronate ester formation (**20**), thereby inducing preassociation of the reactive components. This interaction localizes the sulfonyl fluoride in close proximity to the hydroxamic acid moiety, promoting selective and efficient *O*‐sulfonylation (**21**). As a consequence, the subsequent elimination step leading to isocyanate generation is accelerated, resulting in a substantial enhancement of overall reaction efficiency (Figure 6e).

Building on this design principle, the authors translated the on‐demand isocyanate generation strategy into a ligand‐directed system by developing a stable isocyanate precursor bearing trimethoprim (TMP), a ligand for *Escherichia coli* dihydrofolate reductase (eDHFR) (**18**, Figure 6d). This approach enabled selective and covalent modification of the target protein while maintaining excellent stability under physiological conditions, even in the presence of high concentrations of intracellular nucleophiles such as glutathione. Moreover, incorporation of an alkyne motif into the isocyanate precursor allowed subsequent functionalization via click chemistry, enabling fluorescent labeling with TAMRA (Figure 6e).

Subsequently, Yamatsugu and coworkers performed experimental and theoretical studies on the same isocyanate generation system [62]. They identified the fragmentation of the *O*‐sulfonylated hydroxamate intermediate as the rate‐determining step, which is facilitated by electron donation from the α‐amino group. Structure–activity relationship analyses further supported this mechanistic model. In addition, protein labeling experiments demonstrated that this on‐demand isocyanate system exhibits higher bond‐forming efficiency than photoaffinity labeling (PAL) using diazirine as the reactive warhead [63, 64], highlighting its potential as an alternative covalent labeling strategy under biological conditions.

Collectively, these works establish a ligand‐directed covalent modification strategy in which chemical reactivity is generated on demand at a defined site and time. This expands the design space of ligand‐directed chemistry and complements pre‐encoded warhead strategies such as NASA and ArNASA. However, both these approaches and the induced isocyanate generation systems rely on single‐turnover covalent reactions. In the following section, we discuss advanced ligand‐directed systems based on catalytic proximity control, enabling repeated and programmable modification at target sites and opening a route toward artificial systems for synthetic epigenetics.

## 7 Ligand‐Guided Proximity‐Enabled Catalysis

4‐Dimethylaminopyridine (DMAP) is widely recognized as a nucleophilic acyl transfer catalyst that transiently activates acyl esters and promotes acyl transfer to proximal nucleophilic residues. Hamachi and colleagues extended this classical acyl transfer catalyst into a ligand‐directed context by reporting an innovative strategy in which DMAP was tethered to a carbohydrate ligand [65]. Binding of the carbohydrate ligand with high affinity to the target lectin positioned the DMAP moiety at a defined region on the protein surface, and subsequent addition of acyl donors bearing probes such as fluorescent dyes or biotin induced site‐selective acylation of a nearby tyrosine residue. Indeed, biochemical analyses, including mass spectrometry, revealed that Tyr51 of the animal lectin Congerin II was selectively modified.

Such proximity‐based control of catalytic reactions suggests the possibility of chemically reconstructing PTMs that induce biological outcomes. In living systems, PTMs are intrinsically governed by enzymes that precisely recognize specific target residues and regulate modification states through iterative catalytic cycles. Accordingly, to recapitulate biologically relevant PTMs using chemical catalysts, it is essential to confine reactivity strictly to the vicinity of the intended target site.

**FIGURE 7:**
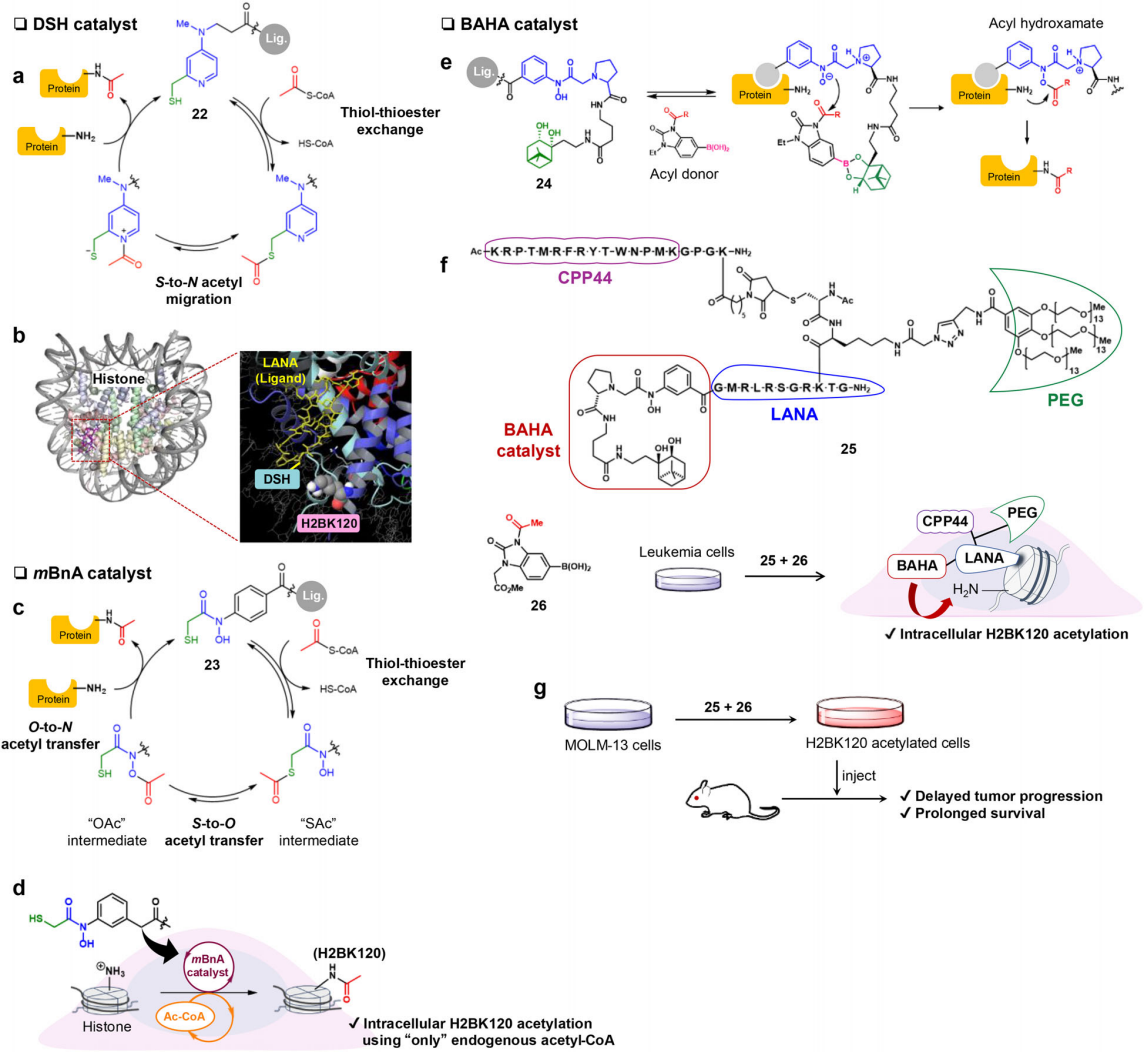
Ligand‐guided, proximity‐enabled chemical catalysis for regioselective histone acetylation and synthetic epigenetic control. (a) Chemical structure of DMAP–SH (DSH) catalyst **22** and schematic of its two‐step acyl transfer mechanism: the thiol captures the acyl group from acyl‐CoA via thiol–thioester exchange, followed by intramolecular transfer to the DMAP moiety, generating a reactive acyl pyridinium intermediate. (b) Site‐selective H2BK120 acetylation using DSH tethered to the *N*‐terminus of the histone‐binding LANA peptide. (c) Structure of *m*BnA catalyst **23** with a hydroxamic acid core, forming a neutral *O*‐acyl hydroxamate intermediate. Mechanism of *m*BnA‐mediated lysine acetylation: the thiol captures the acyl group from acyl‐CoA, followed by intramolecular transfer to the hydroxamic acid, generating a stable intermediate that acylates nearby lysine residues. (d) *m*BnA captures acyl groups from endogenous acyl‐CoAs under physiological conditions, enabling lysine acylation without exogenous donors. (e) Chemical structure and mechanism of BAHA (boronate‐assisted hydroxamic acid, **24**): the nopol diol forms a reversible boronate ester with boronic acid‐containing acyl donors, enriching the local donor concentration. The hydroxamic acid nucleophilically attacks to form a reactive acyl‐hydroxamate intermediate, which transfers the acyl group to nearby lysines. (f) Design of BAHA‐LANA‐CPP44‐PEG catalyst **25** and its application with acyl donor **26** for selective H2BK120 acetylation in leukemia cells. (g) In vivo effects of catalytic H2BK120 acetylation: mice intravenously transplanted with MOLM‐13 cells treated with BAHA‐catalyzed H2BK120 acetylation exhibited delayed tumor progression and prolonged survival.

To address this challenge, Kanai and colleagues focused on histone lysine acylation, a prototypical PTM that is tightly regulated by enzymes. By employing the histone‐binding peptide LANA [66] as a ligand (Figure 7b) and tethering a small‐molecule acylation catalyst to it, they constructed an artificial enzyme‐like reaction environment around the target lysine residue. This approach opened a new research direction—synthetic epigenetics—in which epigenetic modifications can be controlled through purely chemical means [67]. Furthermore, to restrict catalytic activity exclusively to the intended site, acyl coenzyme A (acyl‐CoA), a physiological acyl donor with extremely low background reactivity, was employed as the acyl source. This design effectively suppressed nonspecific acylation across the proteome and enabled catalyst‐driven acyl group transfer to proceed selectively at the target lysine residue.

### 7.1 DSH Catalyst

A breakthrough emerged in 2017 with the development of the DMAP–SH (DSH) catalyst (**22**, Figure 7a) [68]. DSH operates through a two‐step mechanism: the thiol moiety transiently captures the acyl group from acyl‐CoA via thiol–thioester exchange, followed by intramolecular acyl transfer to the DMAP moiety, generating a reactive acyl pyridinium intermediate. When DSH was conjugated to the *N*‐terminus of the LANA peptide, which serves as a histone‐binding ligand, the catalytic center was precisely positioned in proximity to lysine 120 of histone H2B (H2BK120), enabling highly selective acetylation restricted to this specific site (Figure 7b). This study provided the first compelling demonstration that enzyme‐like regioselectivity, as well as histone selectivity, can be achieved solely by constructing an appropriate three‐dimensional arrangement, without reliance on enzymes. The DSH catalyst was subsequently applied to H2BK120‐selective acetylation in living cells. The synthetically introduced epigenomic modification (H2BK120Ac) was stable and not erased by deacetylase activity, thereby reducing the physiological ubiquitination level at the same lysine residue [69].

### 7.2 mBnA Catalyst

Although DSH can activate acyl‐CoAs, DSH alone did not promote histone acetylation by utilizing biogenetically synthesized acetyl‐CoA, which exists in low physiological concentrations (a micromolar range) in living cells due to its moderate catalytic activity. DSH promoted histone acetylation only upon the addition of an exogenous acetyl donor at nonphysiological concentrations exceeding 10 mM [69]. It would be ideal if the catalyst utilizes endogenous acyl‐CoAs to drive regioselective histone acylation, especially for applications to animals. As a step toward a more faithful enzyme mimic, Habazaki, Kanai, and colleagues fundamentally reconceptualized the catalyst design [70]. They abandoned the reliance on the DMAP core due to the following two main reasons: 1) the cationic *N*‐acyl pyridinium intermediate is prone to thiolysis by glutathione (GSH) existing in high concentration (approximately 5 mM) in cells; and 2) DMAP is largely protonated (p*K*_a_ = 8.6) under physiological pH, resulting in diminished catalytic activity. Instead, they adopted a hydroxamic acid (p*K*_a_ = 6.5) framework [71] that generates a neutral *O*‐acyl hydroxamate as the catalytically active species. By precisely tuning the acidity and electronic properties of the hydroxamic acid, they developed the *m*BnA catalyst (**23**, Figure 7c), which efficiently forms the acylated intermediate under physiological acyl‐CoA concentrations in cells.

*m*BnA captures the acyl group from endogenous acyl‐CoAs by its thiol group and converts them into a neutral *O*‐acyl hydroxamate via acyl transfer to its hydroxamic acid core. This intermediate exhibits markedly enhanced resistance to hydrolysis compared with the *N*‐acetyl pyridinium species generated by DSH, thereby maintaining both stability and reactivity under intracellular conditions. As a consequence, *m*BnA is able to promote lysine acetylation solely by using endogenous acetyl‐CoA, without the need for any exogenous acyl donors (Figure 7d). More recently, the catalyst activity was further improved by DFT‐driven electronic tuning of *m*BnA, enabling synthetic perturbation of the in‐cell epigenome solely by the addition of the chemical catalyst [72].

### 7.3 BAHA Catalyst: Synthetic Epigenetic Control of H2BK120 Acetylation in Leukemia Cells

One of the most active acylation catalysts reported to date is BAHA (boronate‐assisted hydroxamic acid, **24**, Figure 7e) combined with bioorthogonal acyl donors [73]. Although this strategy does not exploit endogenous acyl‐CoA, the amount of donor required can be substantially reduced through the donor‐recruiting mechanism. BAHA is characterized by the incorporation of both a hydroxamic acid and a nopol diol moiety. The nopol diol element forms a reversible covalent boronate ester with boronic acid–containing acyl donors, thereby locally enriching the acyl donor concentration in the vicinity of the catalyst. Subsequently, the hydroxamic acid performs a nucleophilic attack to generate a highly reactive acyl‐hydroxamate intermediate, which then transfers the acyl group to nearby lysine residues (Figure 7e).

The BAHA motif was conjugated to the *N*‐terminus of a LANA ligand linked to the leukemia cell‐selective cell‐penetrating peptide CPP44 [69, 74]. In addition, polyethylene glycol (PEG) [69] was appended to protect the LANA ligand from intracellular peptidase‐mediated degradation. Using this strategy, the resulting catalyst **25** and acyl donor **26** were added to the culture medium, enabling Yamanashi, Kanai, and colleagues to achieve H2BK120‐selective acetylation in leukemia cells (Figure 7f) [75]. The synthetic epigenetics mediated by this catalyst in leukemia cells induced selective transcriptional changes. RNA sequencing and RT–qPCR analyses revealed significant upregulation of immediate early genes such as EGR1 and JUN, as well as the cell‐cycle regulator p21. These genes are enriched for binding of the transcriptional elongation inhibitor NELFE, and H2BK120 acetylation promoted dissociation of the NELF complex from chromatin, thereby enhancing RNA polymerase II‐mediated transcription. These transcriptional changes were accompanied by G1/S cell‐cycle arrest and activation of apoptotic pathways. Accordingly, a marked reduction in cell viability was observed across multiple leukemia cell lines, including THP‐1, MOLM‐13, MV‐4‐11, Kasumi‐1, and K562.

Importantly, these effects extended in vivo: mice intravenously transplanted with MOLM‐13 cells subjected to catalytic H2BK120 acetylation exhibited delayed tumor progression and prolonged survival (Figure 7g). Together, these findings indicate that H2BK120 acetylation triggers a coherent cascade—from transcriptional activation of EGR1, JUN, and p21, to cell‐cycle arrest and apoptosis—culminating in robust anti‐leukemic effects at the organismal level.

### 7.4 Synthetic Epigenetic Acetylation of Diverse Histone Lysine Residues

**FIGURE 8:**
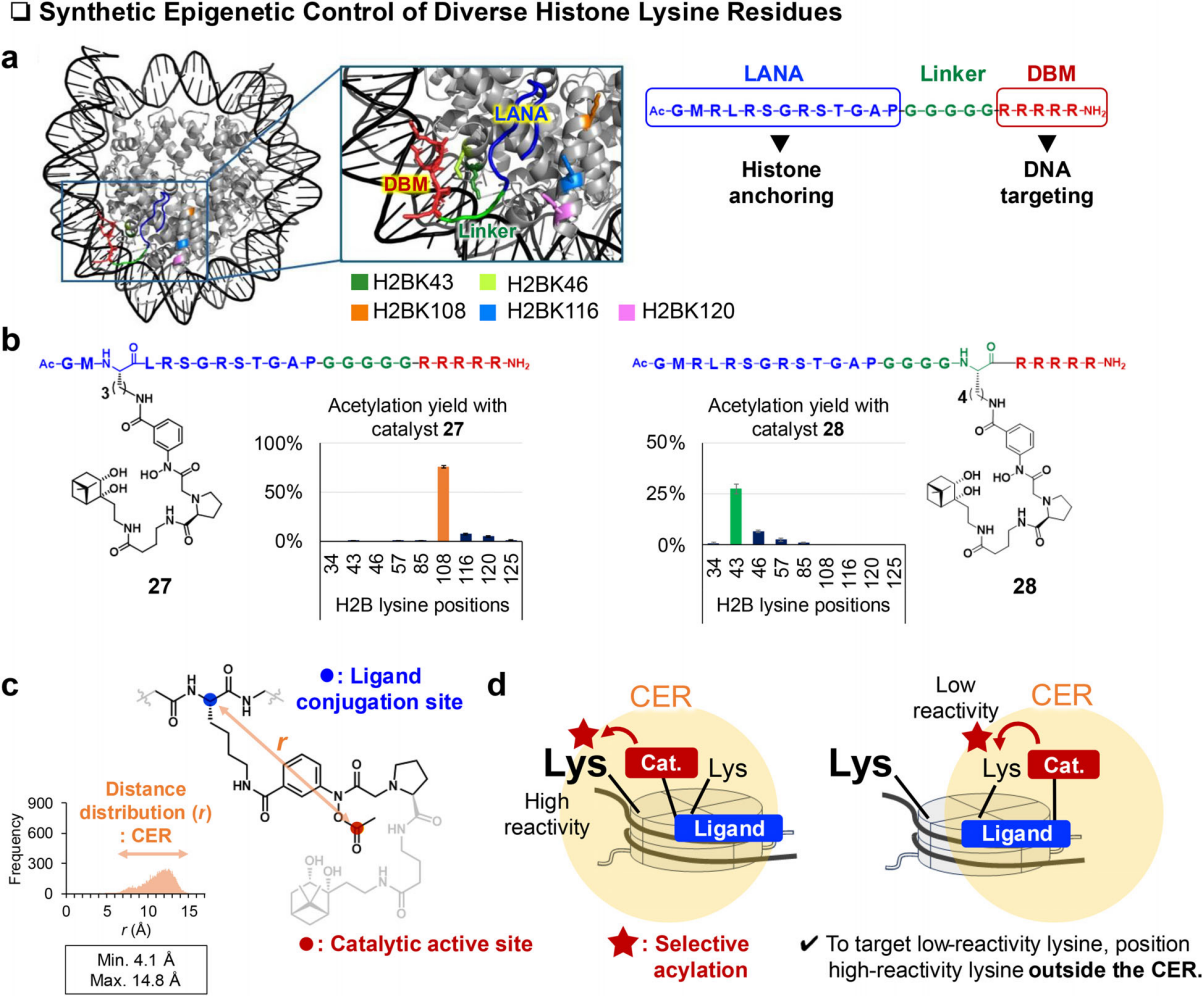
BAHA‐based regiodivergent synthetic acetylation of histone lysine residues. (a) LANA–DBM bivalent ligand structure and binding mode. The ligand consists of LANA and a DNA‐binding motif (DBM). LANA anchors to the histone acidic patch, while DBM interacts electrostatically with DNA, enabling bivalent binding on the nucleosome surface. (b) Regioselective histone lysine acetylation controlled by BAHA positioning on the LANA–DBM scaffold. Catalyst **27**, in which BAHA is installed on a LANA side chain, selectively acetylates H2BK108, whereas catalyst **28**, in which BAHA is positioned in the linker region, targets H2BK43. The graph shows acetylation yields across histone lysine positions, highlighting position‐dependent site selectivity. (c) Definition of the Catalyst Effective Region (CER) based on molecular dynamics simulations. The CER represents the spatial region accessible to the BAHA reactive center and is defined by the MD‐derived distribution of the distance *r* between the catalytic center and the ligand–catalyst attachment point. (d) Model for regioselective acetylation. Lysine modification occurs within the CER. Efficient targeting of low‐reactivity lysines requires positioning highly reactive lysines outside the CER and the target lysine inside it, enabling selective access to the catalytic center.

Nozaki, Onoda, Kanai, and colleagues further expanded the scope of this platform by introducing a modular design that allows systematic tuning of the spatial relationship between the BAHA catalyst and the ligand [76]. A bivalent ligand, LANA–DNA‐binding motif (DBM), which combines LANA with a penta‐arginine DBM, captured a broader region of the nucleosome surface (Figure 8a). In this context, LANA is a peptide that binds to the histone acidic patch with relatively high affinity (*K*_d_ ≈ 160 nM) [77], and is therefore expected to serve as the primary anchoring element for histone recognition. In contrast, the *C*‐terminal cationic DBM interacts electrostatically with the negatively charged DNA phosphate backbone and is likely positioned at energetically favorable DNA‐contact sites. Cooperative engagement of LANA with histones and DBM with DNA thus establishes a bivalent interaction mode, resulting in enhanced overall binding affinity and extended surface coverage of the nucleosome.

Furthermore, installing BAHA at multiple positions within the LANA–DBM scaffold—including the *N*‐terminus, specific side‐chain positions (**27**, which directs acetylation to H2BK108), or the linker region (**28**, which directs acetylation to H2BK43)—enabled selective acetylation not only at H2BK120 but also at these more challenging lysine residues. The structures of the catalysts and their corresponding acetylation yields are shown in Figure 8b. Importantly, this binding architecture increases the accessible surface area of the nucleosome, thereby enabling placement of the BAHA catalyst within the linker region of the LANA–DBM ligand. This design enabled the development of acetylation catalysts capable of targeting lysine residues proximal to DNA, such as H2BK43.

A key conceptual advance emerged from molecular dynamics (MD) simulations, which revealed the spatial reach of the reactive acyl‐hydroxamate moiety of BAHA on the nucleosome surface (Figure 8c). In this analysis, the distance *r* between the acyl carbon of the *O*‐acyl hydroxamate (the reactive center of BAHA) and the ligand backbone carbon was monitored throughout the MD trajectories, yielding a distribution of *r* values spanning 4.1–14.8 Å. This range defines the spatial region sampled by the reactive center during the simulation. Accordingly, the Catalyst Effective Region (CER) is defined as the MD‐derived distribution of *r*, representing the accessible space within which the catalytic center can approach nearby lysine residues. This provides a quantitative framework linking the geometric reach of BAHA to its regioselective acylation behavior [76].

Integration with STP‐alkyne–based reactivity profiling [30] further clarified that H2BK108 possesses inherently high nucleophilicity, whereas H2BK43 is intrinsically unreactive. Based on these findings, the authors proposed that simple inclusion within the CER is sufficient for modifying highly reactive lysines, whereas selective modification of low‐reactivity lysines necessitates the deliberate exclusion of competing high‐reactivity lysine residues from the CER (Figure 8d).

Furthermore, during the development of the H2BK43‐selective acetylation catalyst, lysine side‐chain conformational flexibility emerged as an additional critical determinant of regioselectivity. Although H2BK43 and the adjacent H2BK46 (Figure 8a) exhibit comparable intrinsic reactivity and were both positioned within the CER, selective acetylation was observed exclusively at H2BK43. This preference was attributed to the more structurally constrained side chain of H2BK43, whereas H2BK46 retains greater conformational freedom, indicating that reduced side‐chain flexibility enhances susceptibility to acetylation.

As a result of these studies, artificial histone acetylation catalysts selectively targeting H2BK120, H2BK108, and H2BK43 were successfully established. Together, these findings define three key factors governing the yield and regioselectivity of catalyst‐driven histone acetylation: 1) intrinsic lysine reactivity, 2) geometric proximity to the BAHA motif (i.e., overlap with the CER), and 3) lysine side‐chain conformational flexibility.

### 7.5 Abiotic/Enzymatic Hybrid Catalyst System (ABEHCS)

Liu, Sczepanski, Kanai, and colleagues developed a chemical strategy using tetra‐nucleosome arrays to investigate the relationship between DNA repair and histone modifications in higher‐order oligonucleosomes [78]. Due to difficulties in controlling the spatial arrangement of histone acetylation and DNA damage at the level of nucleosome arrays, previous studies primarily relied on mono‐nucleosomes or uniformly modified chromatin [79].

**FIGURE 9:**
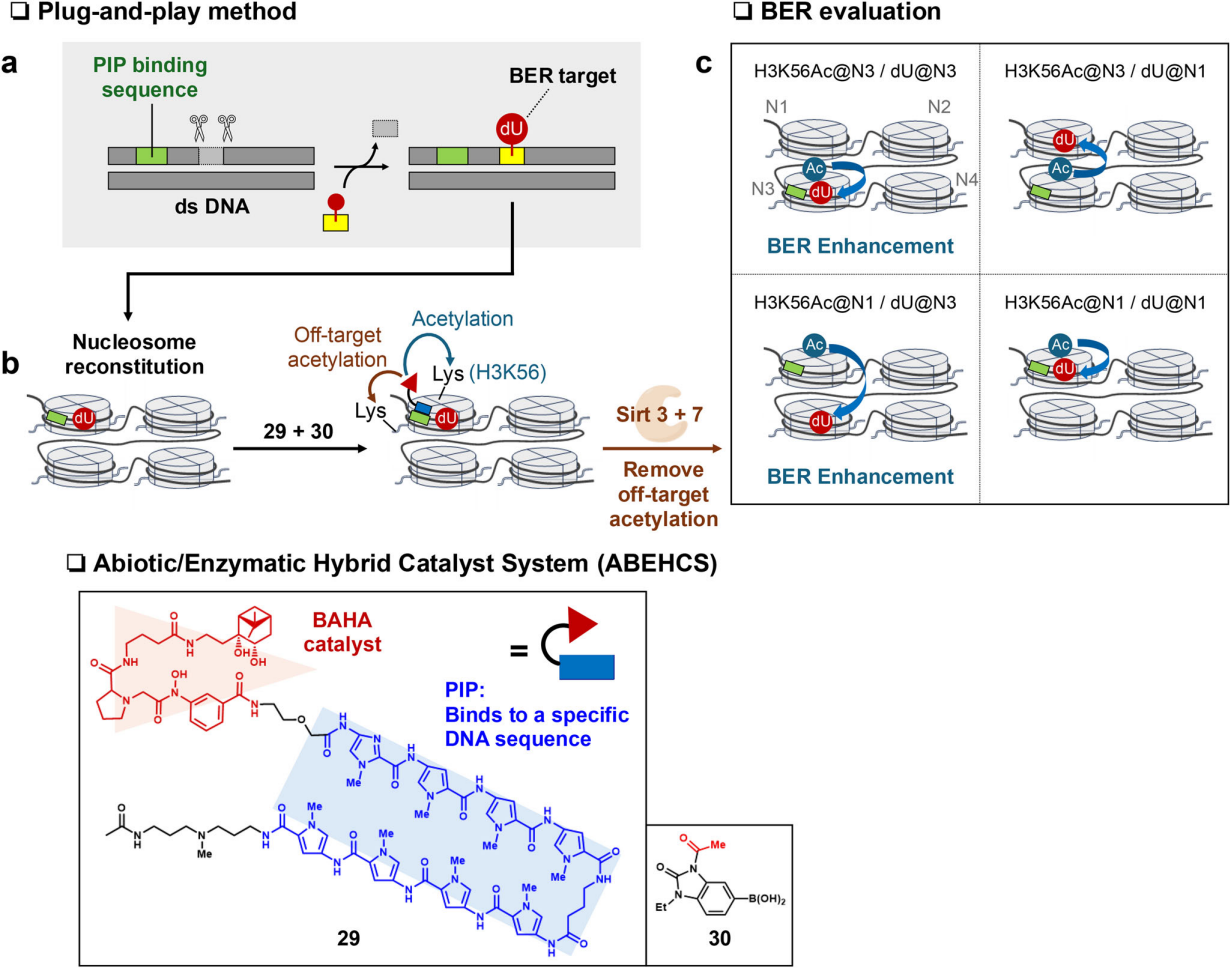
BAHA‐enabled regiodivergent histone acetylation for synthetic epigenome programming in tetra‐nucleosomes using an abiotic/enzymatic hybrid catalyst system, and BER evaluation. (a) A plug‐and‐play strategy enables site‐specific installation of DNA lesions via dU incorporation. (b) In vitro histone acetylation using BAHA‐based catalyst **29** and acetyl donor **30**. Catalyst **29** incorporates a DNA‐binding pyrrole–imidazole polyamide (PIP) that directs acetylation to proximal lysine residues (e.g., H3K56). Subsequent treatment with the deacetylases Sirt3 and Sirt7 removes off‐target acetylation, enriching acetylation at H3K56. (c) BER analysis in tetra‐nucleosome arrays. H3K56 acetylation enhances BER in a position‐ and accessibility‐dependent manner, influenced by the relative positioning of the modification and the DNA lesion. Enhanced repair is observed at structurally less accessible sites (e.g., N3), both when acetylation and damage are located within the same nucleosome and when they reside in distinct nucleosomes.

By combining an abiotic/enzymatic hybrid catalyst system (ABEHCS) with a plug‐and‐play approach, the authors introduced histone acetylation—specifically H3K56Ac, a modification implicated in genome stability [80]—into defined nucleosomes within a tetra‐nucleosome array, while independently positioning DNA damage in the same or different nucleosomes. In the plug‐and‐play method, a short DNA segment at a predetermined position is replaced with a synthetic oligonucleotide carrying a defined lesion (in this study, deoxyuridine (dU)), allowing site‐specific introduction of DNA damage without perturbing the surrounding chromatin structure (Figure 9a) [81]. In the ABEHCS method, BAHA‐containing catalyst **29**, tethered to a DNA sequence‐specific pyrrole–imidazole polyamide (PIP) [82] was used together with acetyl donor **30** to acetylate histone lysine residues in proximity to the target DNA (Figure 9b). In the tetra‐nucleosome array used in this system, the PIP‐binding DNA sequence is present at a single position, which directs catalyst **29** to one nucleosome. Although acetylation of histone H3 at lysine 56 was accompanied by unintended acetylation in histone tail regions, subsequent treatment with Sirt3 and Sirt7 [83] reduced tail acetylation and resulted in modification patterns enriched at H3K56 (Figure 9b). The combined plug‐and‐play and ABEHCS approach enabled the in vitro reconstitution of chromatin environments with non‐uniform distributions of histone modifications and DNA lesions (Figure 9c).

Analysis of the resulting tetra‐nucleosome arrays showed that H3K56 acetylation promotes base excision repair (BER) within nucleosome arrays. This effect depended on the relative positioning of H3K56 acetylation and the DNA lesion, as well as on the structural accessibility of the damaged site. When H3K56 acetylation and DNA damage were located within the same nucleosome, BER enhancement was more pronounced for lesions positioned at structurally less accessible nucleosomes (N3, Figure 9c). In addition, BER promotion was observed when H3K56 acetylation and DNA damage resided in different nucleosomes, provided that the DNA lesion was located at a structurally hindered site.

## 8 Summary and Outlook

Lysine residues serve as central chemical nodes that regulate cellular functions through post‐translational modifications. Local microenvironmental factors, including p*K*_a_, electrostatics, internal hydration, and conformational dynamics, govern the intrinsic reactivity of the ε‐amino group. Studies on buried lysines have revealed that dynamic hydration and fluctuating dielectric environments are dominant determinants of lysine nucleophilicity. Chemoproteomic approaches such as isoTOP‐ABPP have further enabled proteome‐wide visualization of this heterogeneous reactivity landscape. Based on these foundations, diverse strategies exploiting intrinsic lysine reactivity have been developed, including kinetic selection using NHS esters and transition‐state–engineered sulfonyl acrylates. While these methods enable selective lysine modification, they are inherently biased toward the most reactive lysines and therefore remain limited in accessibility to low‐reactivity sites. In contrast, enzymes achieve extraordinary positional selectivity by exploiting not only intrinsic reactivity, but also through three‐dimensional substrate recognition and precise spatial organization. This enzymatic logic has been translated into purely chemical systems through ligand‐directed chemistry. LDAI, NASA, and induced isocyanate generation chemistries demonstrated that proximity or a chemical trigger can gate rapid and highly selective lysine acylation in living cells.

Integrating those concepts into chemical catalysis led to the development of ligand‐guided regioselective histone acylation systems. DSH provided the first proof that artificial catalysts can achieve regioselective histone acylation. *m*BnA further advanced this concept by enabling the use of endogenous acyl‐CoA as the sole acyl donor. In parallel, the BAHA catalyst introduced an orthogonal design strategy based on local enrichment and activation of exogenous boronic acid‐containing acyl donors. The concept of the Catalytic Effective Region (CER) quantitatively links the geometric overlap between the catalyst's spatial reach and a target lysine to regioselectivity, establishing spatial organization as a key determinant of selectivity. In parallel with these developments in chemical catalyst design, an abiotic/enzymatic hybrid catalyst system (ABEHCS) has been reported as a complementary strategy for achieving regioselective histone acylation. ABEHCS combines ligand‐guided chemical acylation mediated by artificial catalysts with subsequent enzymatic deacylation to refine the final modification pattern. By enzymatically proofreading off‐target acylation introduced by the chemical catalyst, this approach enables selective modification of lysine residues that are difficult to access using chemical reactions alone. Further, it allows spatial control of histone modification positions within tetra‐nucleosome arrays.

Looking forward, further generalization to structurally diverse lysines, increased reliance on endogenous metabolites as reaction substrates, and the integration of computational design with experimental catalyst development will be crucial. The reaction pattern should also be broadened beyond acylation. Precision chemistry is thus poised to evolve from a methodological framework for selective modification into a practical chemical platform for programmable regulation and functional manipulation of living systems.

## Conflicts of Interest

The authors declare no conflicts of interest.

## Data Availability

Data is not created in this work.
